# Microwave Cavity Sensor for Measurements of Air Humidity under Reduced Pressure

**DOI:** 10.3390/s23031498

**Published:** 2023-01-29

**Authors:** Alexander Georgievich Galka, Alexander Vladimirovich Kostrov, Stanislav Eduardovich Priver, Askold Vitalievich Strikovskiy, Vladimir Vladimirovich Parshin, Evgeny Alexandrovich Serov, Andrey Sergeevich Nikolenko, Sergey Vladimirovich Korobkov, Mikhail Evgenievich Gushchin

**Affiliations:** Federal Research Center Institute of Applied Physics of the Russian Academy of Sciences, Nizhny Novgorod 603950, Russia

**Keywords:** cavity, dielectric permittivity, amplitude-phase method, water absorption line, absorption line half-width, integral intensity of absorption line, calibration

## Abstract

A high-sensitivity sensor for measuring moisture content in the air or air humidity under low pressure was designed on the basis of a half-wave coaxial microwave cavity. The method of measuring small variations in the signal phase at a cavity excitation frequency of 1.63 GHz was applied to detect low densities of water vapor. It allows the detection of variations in dielectric air permittivity in the seventh decimal place. A prototype of the sensor was tested in a vacuum chamber. It was calibrated by comparing the test results with the readings of a commercial pressure gauge and using the independent method of measuring the moisture content in rarefied air on the basis of the absorption of millimeter waves in the local line of water vapor at 183 GHz. The developed sensor can be used in laboratory experiments and full-scale geophysical research in the atmosphere onboard various aircrafts.

## 1. Introduction

Measuring and monitoring the moisture content in gaseous media including atmospheric air is a paramount scientific and engineering problem, which has been tackled using various methods for several centuries [1,2]. The methods of measuring the water content in gases using microwave devices have been developed since the 1940s [3,4,5,6] due to the advent of required generators, receivers, and circuit solutions [7,8,9]. The microwave methods, especially cavity ones, are characterized by sufficiently high sensitivity [10,11,12], which allows one to develop sensors for the detection of relatively low moisture content [13]. Since microwave humidity sensors do not contain moisture-sensitive materials, they make it possible to detect water vapors with no inertia in real time [14].

The combination of high sensitivity and low inertia in the microwave methods determines the prospects of using microwave sensors for the measurement and monitoring of gas humidity under low pressures. Even a small amount of moisture in a gas can significantly affect the technological processes and the physical phenomena observed in laboratory studies in the field of vacuum technology and engineering [15]. The problems of measuring and monitoring humidity in the atmosphere at various altitudes are of special interest. The effects of the absorption of microwaves in water vapor are extremely important for radioastronomic measurements and, moreover, determine the astronomical climates and locations of the sites chosen for the installation of radioastronomic instruments [16,17]. Water vapor and hydrometeors considerably affect the kinetics of electrons in the electric field and, consequently, determine the evolution and properties of electric discharge phenomena in the atmosphere [18,19,20,21,22,23,24]. Therefore, local measurements of the moisture content in the air, which are performed with reliable and sufficiently small-sized sensors, borne by various vessels in the troposphere and at the stratospheric altitudes, can be of great importance for both geophysical studies and the operability of radio systems.

In this paper, we propose using a microwave cavity sensor based on a half-wave section of a coaxial line to study the moisture content in rarefied gases. Among the various resonant methods, the cavity-based methods for measuring the dielectric parameters of gaseous media, in a sense, have the greatest sensitivity, since the “measuring part” of the sensor is the cavity itself, i.e., the entire volume occupied by the electromagnetic field. At low frequencies (hundreds of MHz), the dimensions of cavity sensors are too large, which makes them inconvenient for practical applications. In addition, at low frequencies, the absolute value of the frequency shift turns out to be smaller for the same permittivity variations, which complicates the measurement procedure and, ultimately, reduces sensitivity. At high frequencies (for example, 10 GHz and above), the size of cavity sensors can be reduced, but at the same time, the circuit solutions become more complicated, and the requirements imposed on the accuracy of manufacturing and adjusting device elements become stricter. A trade-off solution is a cavity sensor operating in the decimeter wavelength range and with a characteristic size of approximately 10 cm. On the one hand, such a sensor is quite small in size; on the other, it combines an acceptable sensitivity with ease of manufacture. The shift in the cavity eigenfrequency is determined by the pressure-dependent dielectric permittivity *ε* of the gas in the cavity. Under a low gas pressure ranging from zero to several Torr, the value of *ε* differs from unity in the sixth or seventh decimal place. The relative changes in the cavity eigenfrequency, which should be detected, turn out to be of the same order of magnitude. In order to detect minor frequency shifts within the limits of the resonance curve, the amplitude-phase method developed earlier in [25] is used in this paper. The experiments were performed at IAP RAS in the vacuum chamber of the Sprite setup [26].

## 2. Description of the Humidity Sensor

**Figure 1:**
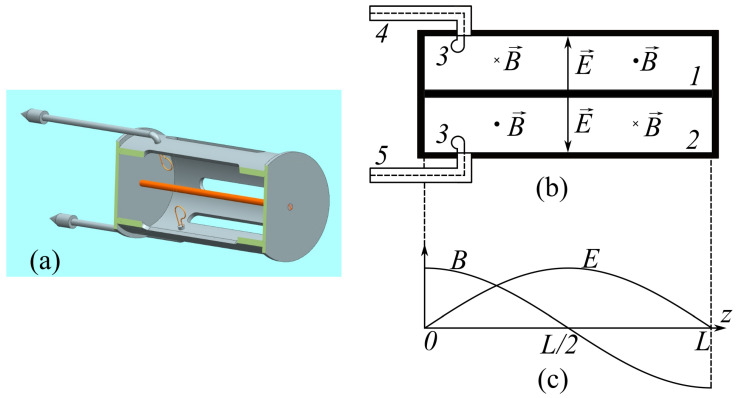
Humidity sensor. (**a**) Sectional view of the 3D model; (**b**) cavity diagram: internal conductor (1), external conductor (2), magnetic coupling loops (3), cavity excitation line (4), and receiving line (5); and (**c**) distribution of electric E and magnetic B fields along the axis *z* of the cylinder.

The chosen design ensures sufficiently high mechanical rigidity and stability of the sensor parameters under external influences (thermal, mechanical, etc.). While having the same dimensions, half-wave cavity sensors have a number of advantages over quarter-wave sensors (as described, e.g., in [14]), including a higher operating frequency and a higher quality factor due to lower radiation losses, which, in turn, allows one to increase the sensitivity. We used several variants of microwave cavity prototypes with operating frequencies of 1 through to 2 GHz. The sensor used mainly in the experimental studies was a half-wave coaxial cavity with the length *L* = 95 mm, which was short-circuited with copper plates at both ends. The design of the sensor is shown in Figure 1. The internal (1) and external (2) conductors of the cavity had the diameters $d_{1}=$ 4 mm and $d_{2}=$ 45 mm, respectively. The mass of the sensor was 320 g. High-frequency oscillations were excited, and the resonance responses were received by means of magnetic coupling loops (3) of 6 mm in diameter, which were installed on the internal surface of the external conductor near one end of the cavity. At a pressure below 0.01 Torr, the eigenfrequency of the cavity was $f_{0}=$ 1632.6 MHz, the resonance curve bandwidth was 4.2 MHz, and the Q-factor was *Q* = 387. Estimations show [25] that the main contribution to the total Q-factor of the measuring system is made by the energy losses determined by the magnetic coupling of the cavity with excitation line (4) and signal reception line (5). Magnetic coupling loops are the simplest solution for cavity excitation and detection of the cavity response using coaxial cable lines. In this case, the best coupling with the cavity is achieved by placing magnetic coupling loops at the short-circuited ends of the cavity, i.e., in the magnetic field antinodes. The specific positions and sizes of the magnetic coupling loops are chosen empirically by ensuring the maximum cavity Q-factor, on the one hand, and an acceptable cavity response amplitude, on the other hand.

In the coaxial cavity, the TEM mode was excited at the first harmonic of the fundamental frequency. The electric field is at a maximum in the middle part of the cavity and is equal to zero at the short-circuited ends (Figure 1c). The cavity sensor was operated in the continuous wave (CW) regime at the fundamental frequency of the cavity.

To let a gas into the sensor, eight narrow cuts were made in the side wall along the axis of the cylinder. They ensure weak modifications of the structure of the surface electric currents when the cavity is excited in the microwave range. The length and width of each cut were 55 mm and 5 mm, respectively. The transverse size of the region of localization of the high-frequency field out of the cavity was small and close to the width of the cut by the order of magnitude, which allowed us to exclude the influence of external objects on the readings of the instrument.

In several experiments, a different cavity sensor was used, which had a lower resonance frequency, $f_{0}=$ 1031.6 MHz, and a slightly higher Q-factor, *Q* = 450 [27]. The length of the second sensor was L= 150 mm, and the diameters of the internal and external conductors were $d_{1}=$ 5 mm and $d_{2}=$ 20 mm, respectively.

Recall the method of measuring the parameters of a gas media using microwave cavity sensors. When a gas with dielectric permittivity *ε* enters the sensor, the eigenfrequency *f* of the cavity formed by a section of a coaxial line decreases in accordance with the following expression [28]:

(1) $$f=f_{0}/\sqrt{\varepsilon }.$$

An advantage of microwave cavity modes when measuring the parameters of gaseous media is the possibility to measure small-scale differences in the dielectric permittivity of gases from unity, Δε=ε−1. At identical partial pressures, the correction Δε for water vapor proves to be significantly greater in the microwave range (up to one and through to two orders of magnitude) than the corrections for the majority of the gases that comprise air, for example. This fact makes it possible to measure gas humidity based on the changes in the resonance characteristics of microwave sensors.

The frequency shifts to be measured can be estimated on the basis of the tabular and graphic data about the values of the dielectric permittivity of dry air and water vapor (*ε*_*air*_ and *ε*_*H2O*_, respectively) at a temperature of 300 K [2,6,29]:

(2) $$\begin{array}{c} \varepsilon_{air}=1+7.2\cdot 10^{-7}\cdot P,\\ \varepsilon_{H2O}=1+1.25\cdot 10^{-5}\cdot P, \end{array}$$

where P is the gas pressure in Torr. Under a change in the water vapor pressure *P* by 1 Torr, the characteristic value Δε=ε−1 of variations in the dielectric permittivity has an order of magnitude of 10^−5^. The corresponding cavity frequency shift at *f*_0_ ~ 1 GHz is a small quantity, $\left|\Delta f\right|=\left|f-f_{0}\right|\;\sim \;10$ kHz. These frequency shifts |Δf|, which are very small relative to the resonance width (~5 MHz), are hard to determine using the method based on the shift in the maximum of the resonance curve. Therefore, we used the amplitude-phase method to measure small values of Δf.

## 3. Amplitude-Phase Method of Measuring Small Frequency Shifts

The output signal is proportional to the phase difference ∆φ between the reference signal and cavity response signal $U_{0}$. The amplitude-phase method uses the dependence of the phase difference Δφ on the gas humidity inside the cavity [30]. The phase shift is determined by the variation in the dielectric permittivity Δε=ε−1. The variation Δε is determined by the presence of gas in the cavity, which, in turn, depends on the pressure of water vapors: Δε=Δε(P). According to [25], the phase shift for the signal at a frequency equal to the central frequency of the cavity in vacuum, *f*_0_, satisfies the relationship that has the form tg(Δφ)=Q·Δε/2. Proportional (linear) measurements of the correction Δε for the dielectric permittivity correspond to the measured small pressures *P*. Thus, the value of the phase shift proves to be proportional to the pressure of the gas (water vapor).

**Figure 2:**
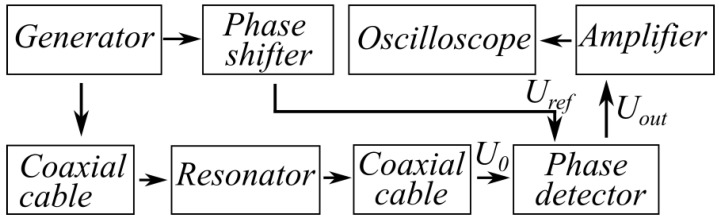
Block diagram of amplitude-phase humidity measurements.

In our experiments, a resistive element divided the signal generated at the eigenfrequency $f_{0}$ of the cavity, which had a frequency stability of at least $10^{-8}$, into 2 arms (Figure 2). The probing signal $U_{0}$, which was transmitted through the gas-filled cavity and a line with a variable length, was mixed with the signal $U_{ref}$ of the reference arm. The phase detector was based on the ADE-20 [31] chip operating in the 1.5–3.1 GHz range with an attenuation coefficient of 6 dB at the operating frequency *f*_0_ = 1.63 GHz.

The signal produced by the phase detector was amplified using a low-frequency amplifier and then sent to the input of the digital oscilloscope. The signal $U_{out}$ at the output of the phase detector was determined by the amplitude of the signal transmitted through the cavity and the phase difference Δφ between the signals $U_{0}$ and $U_{ref}$. The variable-length line acted as a phase shifter (see Figure 2) and was adjusted in such a way that the phase shift Δφ between the reference signal and the response signal of the cavity in the absence of a gas inside it (in vacuum) was equal to zero. Accordingly, $U_{out}=0$ in the absence of a gas inside the cavity. 

**Figure 3:**
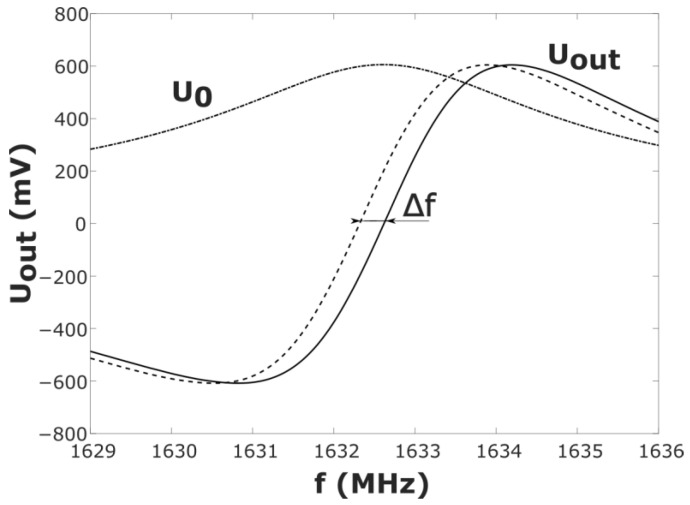
Experimental characteristics of the humidity sensor. $U_{0}\left(f\right)$ is the amplitudefrequency dependence of the signal transmitted through the cavity, and $U_{out}\left(f\right)$ is the frequency dependence of the signal output from the measuring system for a sensor with and without the gas (the dashed line and the solid line, respectively).

After zero-level compensation, the output signal from the phase detector can be represented as $U_{out}=k\cdot U_{0}\cdot \sin\left(\Delta \varphi \right)$, where $U_{0}$ is the voltage amplitude of the cavity response signal, and k is a dimensionless factor. This k factor takes into account all types of losses in the high-frequency signal at the measuring line as well as the amplification of the low-frequency signal in the phase detector circuit. The value of the factor can be found by calibrating the sensor without a gas in the cavity (in vacuum). The experimentally obtained dependence of the output signal from the phase detector on the frequency $U_{out}\left(f\right)$ in the 1629 MHz < f < 1636 MHz range is shown in Figure 3. Note that injection of a gas (water vapor) with a dielectric permittivity close to unity into the cavity does not lead to a variation in the calibration coefficient *k*.

**Figure 4:**
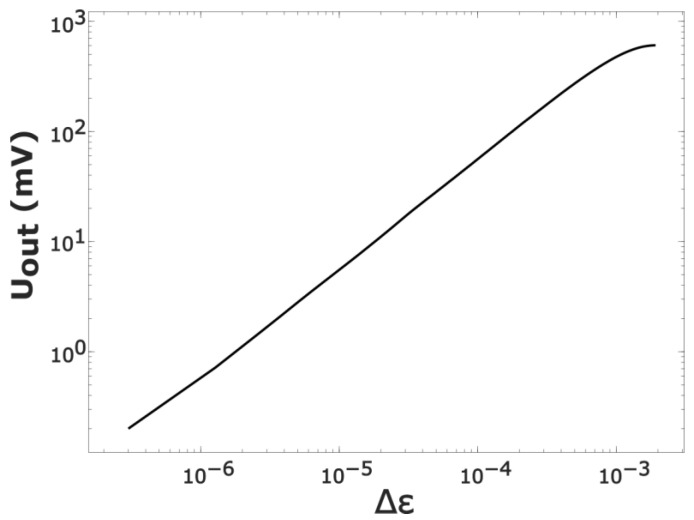
Output signal as a function of the variation Δ*ε* in the dielectric permittivity of the medium inside the cavity. The zero signal from the phase detector corresponds to the equality Δε = 0 (gas-free cavity).

When a gas with dielectric permittivity *ε* entered the sensor cavity, the curve $U_{out}\left(f\right)$ moved down the frequency (Figure 3, the dashed line). It follows from Equation (1) that the equation δf=−0.5·Δε relates the relative frequency shift $\delta f=\Delta f/f_{0}$ with Δ*ε* at Δ*ε* ≪1. Thus, in accordance with $U_{out}\left(f\right)$ (Figure 3), one can easily obtain the output characteristic $U_{out}\left(\Delta \varepsilon \right)$ of the measuring system (Figure 4): $U_{out}\left(\Delta \varepsilon \right)=U_{out}\left(f_{0}+\delta f\right)-U_{out}\left(f_{0}\right)$.

The dependence $U_{out}\left(\Delta \varepsilon \right)$ allows one to find the dielectric permittivity *ε* of the gas inside the cavity and use Equation (2) to determine the connection between the pressure and the voltage at the detector output $U_{out}=b\cdot P$, where the proportionality factor (sensitivity) *b* depends on the type of gas. With expressions (2) taken into account, the following values of the sensitivity can be expected for the actual parameters of the design used in the experiments:

(3) $$\begin{array}{c} b_{air}\approx 0.4\;mV/Torr\;(dry\;air)\\ b_{H2O}\approx 6.8\;\;mV/Torr\;(water\;vapor). \end{array}$$

In other words, the sensor signal is the output voltage of the phase detector proportional to the phase shift between the response signal of the gas-filled cavity and the reference signal, which is, in turn, proportional to the small variation in the dielectric permittivity of the gas compared with unity (or vacuum).

## 4. Calibration Procedure of the Humidity Sensor

The humidity sensor was installed in a metal chamber of 2 m long and 1.6 m in diameter [26], which was evacuated using a fore vacuum pump to the final vacuum at a pressure of $3\cdot 10^{-2}$ Torr. This pressure of the residual gas proved to be lower than the sensitivity level of the measuring system. The pressure *P* in the chamber was monitored with a PfeifferPKR251 vacuum meter in the range from $10^{-3}$ to 10 Torr. High values of *p* > 10 Torr were recorded with a DV 05100 device [32]. The temperature in the chamber was constant and equal to 23±0.1 °C. The atmospheric air was tapped from the working room. The relative humidity of the air, at which the measurement took place, was 23% under natural conditions. The absolute moisture content was 5 g/m^3^ under atmospheric pressure. The pressure *P* in the chamber, which was determined based on the readings of the vacuum sensor, varied in the range from 0.1 to 270 Torr.

The spectroscopic method [33,34], which has been used successfully for absolute humidity measurements [35], was used for the physical calibration of the humidity sensor. Analysis of the line profile recorded by means of a classic video spectrometer makes it possible to determine both the partial pressure of the water vapor and the partial pressure of the buffer gas (e.g., air) in the two-component mixture.

**Figure 5:**
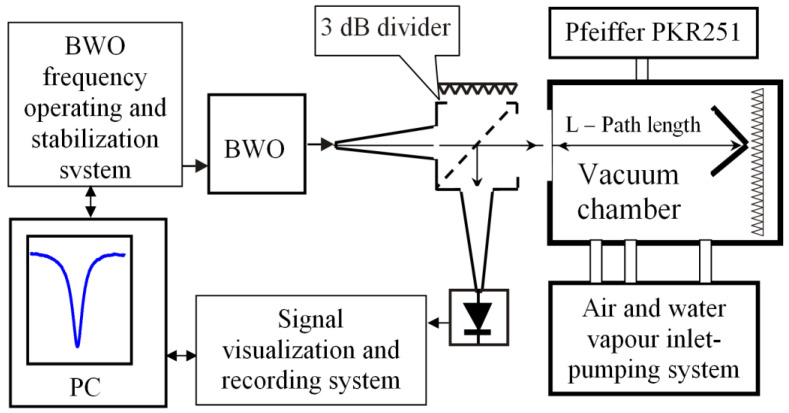
Scheme of the setup for spectroscopic measurements in the frequency band near 183 GHz.

The concentration of water molecules in the considered volume of the gas mixture was determined on the basis of analysis of the parameters (intensity and resonance width) of the water vapor absorption line near 183 GHz. For this purpose, a path of approximately 1 m long was arranged in the vacuum chamber, and a quasi-optical beam of electromagnetic waves with an appropriate frequency was propagated along this path. A horn converted the backward-wave oscillator (BWO) radiation from the waveguide in the main cross-section into a quasi-optical beam (Figure 5), which was injected into the considered volume through a vacuum window with antireflection corrugations [36]. The parameters of the OV-86 BWO were as follows: the frequency range 118–190 GHz, the maximum power 40 mW, and the supply voltage 500–1900 V. The beam reflected from the corner reflector installed in the considered volume was sent to a similar receiving horn via a 3-dB diplexer and then reached a detector based on a diode with a Schottky barrier. The detected signal was fed into the oscilloscope, digitized, and recorded in the computer memory for further analysis.

**Figure 6:**
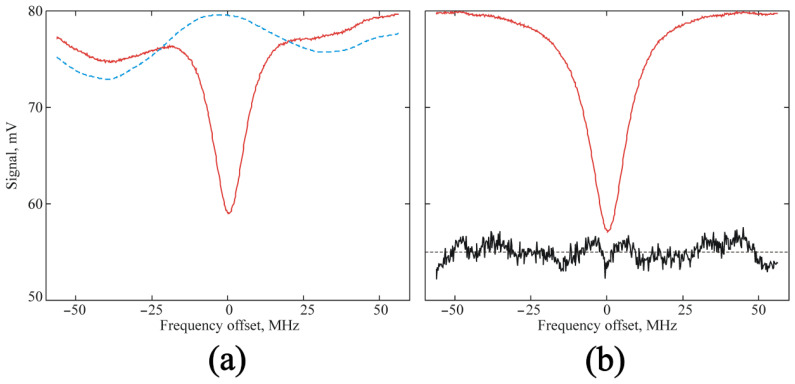
Experimental recording of the H_2_O line near 183 GHz, which was obtained under a pressure of 0.43 Torr: (**a**) the initial recording averaged over 16 triangular scans (solid red line) and the base line (dotted blue line); (**b**) the profile obtained after the subtraction of the variable component of the baseline (solid red line) and the residual “experiment minus model” shifted to 55 mV relative to 0 and multiplied by 10.

The BWO frequency was scanned obeying the triangular law in the vicinity of the resonance absorption line. Therefore, the oscilloscope, whose scanning was synchronized with the BWO scan, displayed the actual pattern of the absorption line against the background of the frequency dependence of the BWO power (baseline) (Figure 6a). The baseline was recorded after the minimum pressure in the vacuum chamber was achieved before the start of feeding in the studied gas. After that, the variable component was subtracted from the obtained line recordings (Figure 6b).

The detected signal is proportional to the power fed to the detector, S = λP = λP_0_(f)∙exp(–α(f)∙L), where α(f) is the coefficient of absorption in the gas at the frequency *f*, and *L* is the length of the path. Then, the natural signal logarithm is ln(S) = ln(λ) + ln(P_0_(f)) – α(f)∙L. On top of the frequency dependence of the power, the form of the line is also distorted due to the fact that a small fraction of the source power travels over a distance exceeding *L* due to parasitic reflections. Assuming that the parasitic reflections are low, and the frequency dependence of *P*_0_(*f*) is weak near the line center f = f_0_, we obtain

ln(S) ≈ C_0_ + C_1_∙(f − f_0_) + C_2_∙(f − f_0_)^2^ − α(f)∙L∙(1+C_3_∙(f − f_0_)). (4)

Here, α(f) is the Voigt profile, which describes the form of the water vapor line, and Ci are the adjustable parameters that determine the frequency dependence of the source and the distortion of the line profile due to parasitic reflections. Fitting the model using the least-square method, we determined the half-width and integral intensity of the line. The integral intensity depends only on the partial pressure of water vapor, and the half-width is determined by the partial pressure of other gases (air) as well. Thus, a significant advantage of using the spectroscopic method for the determination of the partial pressure of water vapor is that it is selective with regard to the composition of the gas mixture. Contrasted with the capacity, thermocouple, or cavity sensors, in this case, the result of determining the pressure via the integral line intensity is independent of the partial pressure of other components of the gas mixture, unless they have strong absorption lines near 183 GHz. This method for humidity measurement is characterized by high sensitivity since the power reduction at the line center is approximately 30% for a total path length of 286 cm up to water vapor pressures of approximately 0.05 Torr. At a lower pressure, the line amplitude starts decreasing due to the transition from the collisional broadening regime to the Doppler one.

The error in determining the partial pressure of water vapor depends on several factors. First, it includes the error in the intensity of the water vapor line. In [37], the intensity of the 183 GHz line was measured with an accuracy of approximately 1%. The second error in measuring the length of the path, along which the absorption occurs, is approximately 0.1%. The 3rd component is the statistical error in fitting the model parameters of 0.5% for the integral intensity in the range of water vapor pressures from 0.02 to 1.1 Torr.

## 5. Results and Discussion

**Figure 7:**
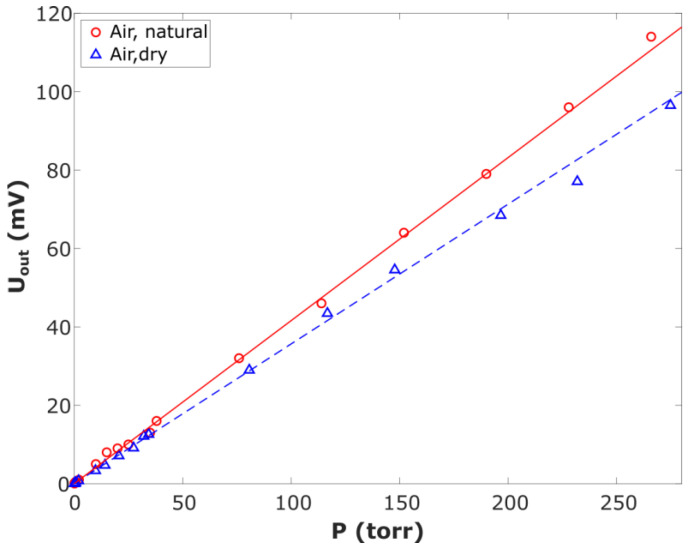
Output signal of the humidity sensor as a function of the air pressure: ○ is for air under natural conditions, and Δ is for dry air with a humidity of less than 1%.

When the air was pumped into the vacuum chamber, the signal $U_{out}$ from the cavity sensor was recorded. The experimental data obey the linear law (Figure 7, the solid and dashed lines) with the proportionality coefficient, which is *b*_air_ = 0.35 mV/Torr for dry air and 0.45 mV/Torr for humid air, with a good degree of accuracy. The difference between the experimentally measured *b*_air_ and the value (4) calculated for dry air was not more than 15%. Earlier, we obtained a similar result using a similar design of the sensor with the operating frequency $f_{0}$ = 1.03 GHz and the Q-factor of the cavity Q ~ 450.

**Figure 8:**
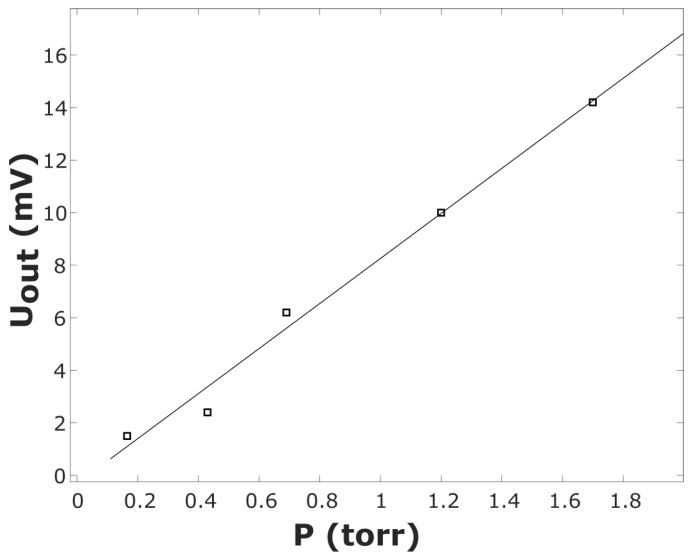
Output signal of the humidity sensor as a function of the vacuum meter readings during water vapor injection.

When water vapor was injected into the vacuum chamber from a previously evacuated flask with distilled water, the range of variations in the pressure *P* was 0.2–1.7 Torr. In this case, the observed signal $U_{out}$ (Figure 8, □) was an order of magnitude greater than that in the case of injection of air (Figure 7, Δ), while the vacuum meter readings were identical. This can be explained by a significant difference in *ε* for air and water vapor in accordance with (2). The solid line in Figure 8 shows the linear approximation $U_{out}=8.5\cdot P$. The actual value of the sensitivity (coefficient of proportionality between $U_{out}$ and P) turned out to exceed the value estimated via Formula (2) by 20%. This fact justifies the use of the calibration procedure for the experimental humidity sensor.

**Figure 9:**
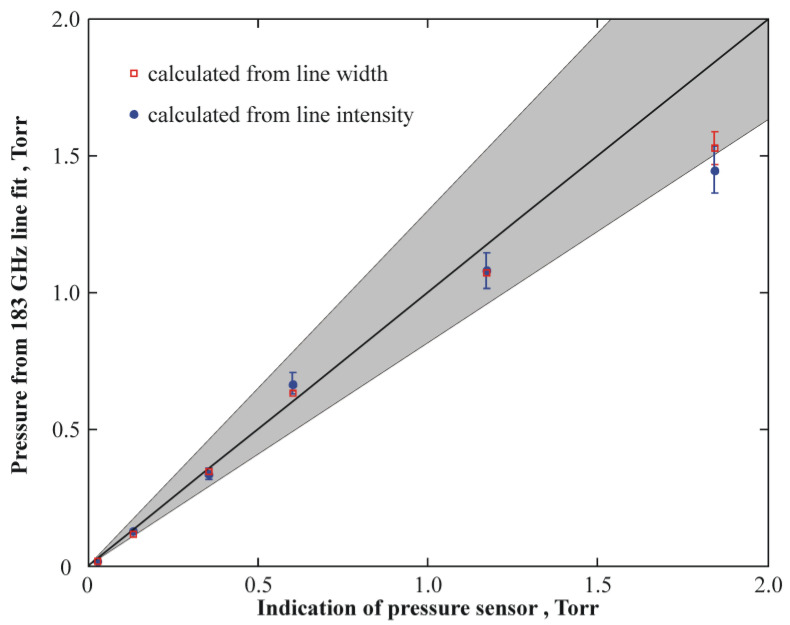
Experimentally determined water vapor pressure as a function of the vacuum gauge readings. The data are obtained using the spectroscopic method. The grey area shows a possible deviation of the real pressure from the readings of the vacuum gauge with the gauge error taken into account.

Synchronous measurements were performed by using the independent spectroscopic method to monitor the measurements of the absolute humidity made by the cavity sensor. Below, the pressure *P*_*H2O*_ of water vapor, which was determined using the spectroscopic method, is presented as a function of the vacuum gauge readings *P* (Figure 9). The range of the measurements of the water vapor pressure *P*_*H2O*_ was 0.13–1.44 Torr.

The results obtained on the basis of both the ratio of the absorption line half-width and the self-broadening coefficient (Figure 9, □) and the integral intensity (Figure 9, *○*) nearly coincide, which confirms that only water vapor was present in the vacuum chamber. The shadowed region in Figure 9 corresponds to the measurement error of the vacuum gauge, which is equal to 30%.

Basing on the data shown in Figure 8□ and Figure 9, the humidity cavity sensor was calibrated. Within the framework of this procedure, the absolute value of the water vapor pressure *P*_*H20*_ was found using the spectroscopic method and specified for each $U_{out}$ reading.

Synchronous humidity measurements made in this range allow one to measure humidity with good accuracy at a level of 20–30%.

The significance of the cavity method proposed for measurements of the moisture content at low pressures was determined via both scientific and engineering tasks and the possibility to calibrate the device using the independent spectroscopic method that yields the absolute content of water vapor under pressures below 10 Torr.

Under controlled external pressure and temperature conditions, the sensor can be used conveniently as a routine humidity control instrument in the laboratory modeling of electric discharge phenomena in the Earth’s atmosphere, including air ionization in the field of the electromagnetic pulse [38] and streamer discharges [39]. In geophysical sensor applications, one should allow for the influence of temperature on the device readings due to the changes in the geometric dimensions of the cavity. Then, it is necessary to compensate additionally for the temperature drift of the sensor body and introduce the correction factor when determining the eigenfrequency of the cavity. The temperature drift can be compensated by adding a second vacuum-tight cavity, whose size is identical to the size of the initial sensor, in the circuit of the reference signal $U_{ref}$ (Figure 2). The second cavity should be evacuated to a pressure below 0.1 Torr corresponding to the air sensitivity threshold. Then, the frequency shifts of the first and second cavities, which are determined by thermal expansion or compression and, correspondingly, the phase shifts in the measuring and reference lines, will be identical and compensated in the phase detector. The output signal $U_{out}$ will not depend on the variations in the geometric dimensions of the sensor. It should be noted that the potentialities of the developed method are not limited by measurements of the stationary value of the water vapor pressure. The sensor’s speed of response, which is determined by the cavity Q-factor, allows one to detect humidity fluctuations with the submicrosecond time resolution being much less than the characteristic mass transfer times in gas media (milliseconds and longer).

## 6. Conclusions

A humidity sensor was developed for the determination of the water vapor content in rarefied gases. The sensor is a high-Q cavity with an eigenfrequency of 1632.6 MHz in vacuum, which is based on a half-wave section of the coaxial line. The eigenfrequency of the cavity depends on the dielectric permittivity of the gas, and the difference in this parameter from unity is proportional to the pressure at a fixed temperature. Small frequency shifts were detected by observing the amplitude-phase characteristics of the diagnostic system. The dependence of the output voltage on the gas pressure was obtained.

The sensor was tested in a large-scale vacuum chamber with a volume of 4 m^3^. The linear dependence of the output signal on the pressures of dry and wet air in the range from 0.1 to 270 Torr was obtained in the process of filling the cavity with air. The experimental data agree with the calculation results with an accuracy of approximately 15%. When the chamber was filled with water vapor, the cavity sensor was calibrated using the spectroscopic method based on the measurement of the absorption of millimeter waves in the local line of water vapor at 183 GHz. As a result, the linear dependence of the output voltage of the sensor on the water vapor pressure was obtained in the pressure range from 0.1 Torr to 1.4 Torr.

## Data Availability

The data presented in this study are available on request from the corresponding author.
